# Supplementary material for: Xylan Decomposition in Plant Cell Walls as an Inducer of Surfactin Synthesis by Bacillus subtilis
Source: Biomolecules. 2021 Feb 8;11(2):239. doi: 10.3390/biom11020239 (PMC7915361; doi:10.3390/biom11020239)
Supplement: Supplementary file 1 [file biomolecules-11-00239-s001.pdf]

Supplementary material

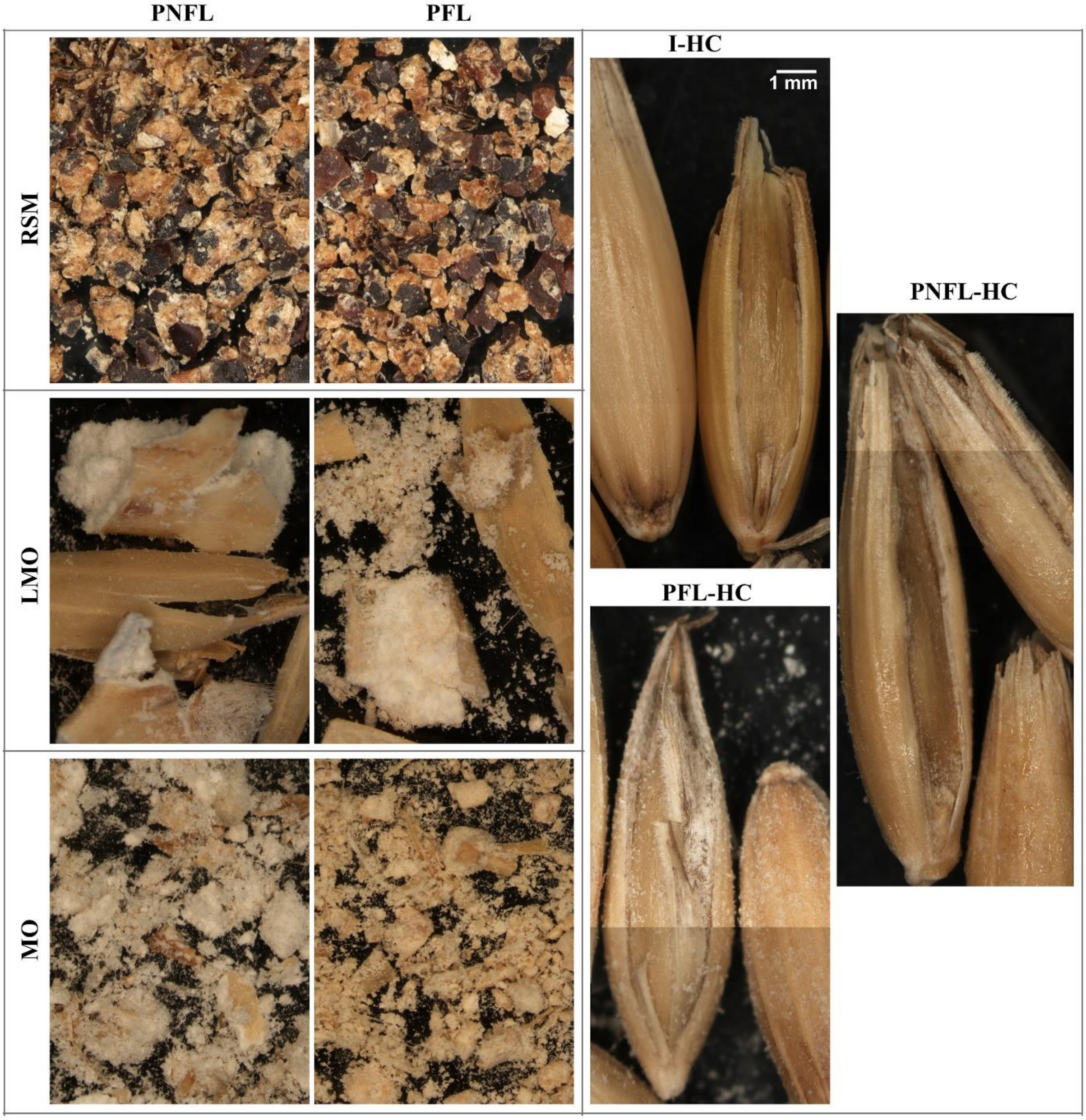

Supplemental Figure S1. Four types of plant material was used in solid state fermentation (SSF): rapeseed meal (RSM); oat hulled caryopses (HC); large fragments of milled oats (LMO); and milled oats (MO) after removal of the large fragments. The biomass was: pasteurized, fermented and lyophilized (PFL); pasteurized and lyophilized (PNFL); or remained intact (I). All images were taken using the stereomicroscope and are shown at the same magnification.

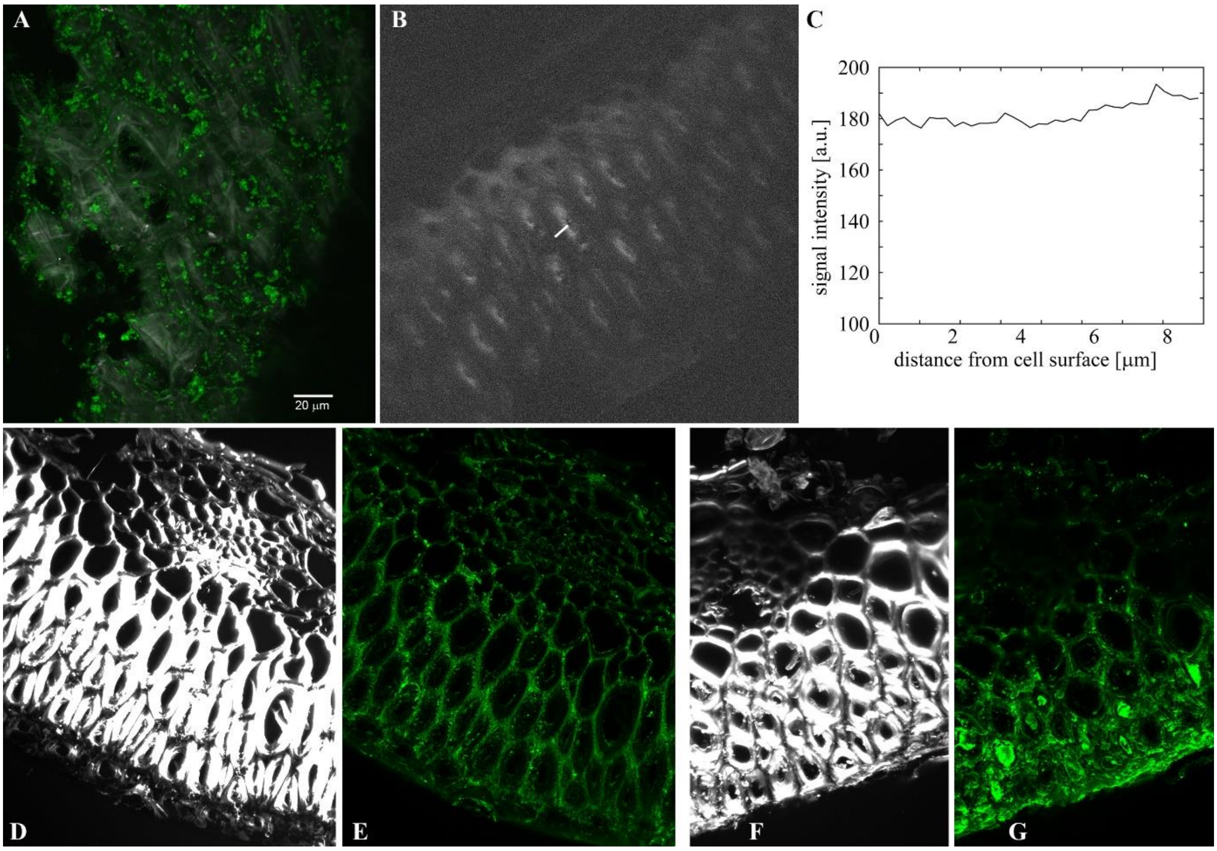

Supplemental Figure S2. Confocal images of MO (**A**) and lemma cross-sections (**B-G**). Z-projections of maximal signal from confocal stacks were obtained in Fiji. **A.** Blind control of immunolabelling (primary antibody omitted) of MO shows secondary antibody (green channel) attachment to starch grains. Cell walls were stained by Calcofluor White (grey channel). **B-C**. Auto-fluorescence of lemma cell walls obtained with the same microscope settings as those used for observation of stained samples shown in Fig. 7. In C, the signal intensity is plotted along the transect marked with white line segment in B. **D-G.** Calcofluor White (grey channel) and LM11 (green channel) signals in cross-sections of I-HC (**D-E**) and PFL-HC (**F-G**) lemma. In I-HC section LM11 signal co-localizes with primary cell walls while in PFL-HC some of the signal may be an artefact. All images are shown at the same magnification.
